# Unravelling the In Vitro and In Vivo Anti-*Helicobacter pylori* Effect of Delphinidin-3-*O*-Glucoside Rich Extract from Pomegranate Exocarp: Enhancing Autophagy and Downregulating TNF-α and COX2

**DOI:** 10.3390/antiox11091752

**Published:** 2022-09-04

**Authors:** Amany E. Ragab, Lamiaa A. Al-Madboly, Ghada M. Al-Ashmawy, Maha Saber-Ayad, Mariam A. Abo-Saif

**Affiliations:** 1 Department of Pharmacognosy, Faculty of Pharmacy, Tanta University, Tanta 31527, Egypt; 2 Department of Pharmaceutical Microbiology, Faculty of Pharmacy, Tanta University, Tanta 31527, Egypt; 3 Department of Biochemistry, Faculty of Pharmacy, Tanta University, Tanta 31527, Egypt; 4 Department of Clinical Sciences, College of Medicine and Research Institute for Medical and Health Sciences, University of Sharjah, Sharjah 27272, United Arab Emirates; 5 Department of Pharmacology, College of Medicine, Cairo University, Giza 11956, Egypt

**Keywords:** Pomegranate, anthocyanin, *H. pylori*, Malondialdehyde, catalase, TNF-α, COX2, autophagy, *Beclin1*, *ATG5*, *ATG12*

## Abstract

Fruits containing antioxidants, e.g., anthocyanins, exhibit antimicrobial activities. The emergence of drug resistance represents a major challenge in eradicating *H. pylori*. The current study aims to explore the effect of pomegranate exocarp anthocyanin methanol extract (PEAME) against *H. pylori* isolates recovered from antral gastric biopsies. The UPLC-PDA-MS/MS and ^1^H NMR analyses indicated delphinidin-3-*O*-glucoside as the major anthocyanin in the extract. The PEAME showed activity against all tested resistant isolates in vitro recording minimum inhibitory concentration (MIC) and minimum bactericidal concentration (MBC) values of 128 and 256 µg/mL, respectively. In vivo investigation included evaluation of the rat gastric mucosa for malondialdehyde (MDA), catalase activity, COX2, TNF-α, and key autophagy gene expression. The combination of pomegranate with metronidazole markedly reduced the viable count of *H. pylori* and the level of COX2, with alleviation of *H. pylori*-induced inflammation and oxidative stress (reduction of MDA, *p*-value < 0.001; and increase in catalase activity, *p*-value < 0.001). Autophagy gene expression was significantly upregulated upon treatment, whereas TNF-α was downregulated. In conclusion, we comprehensively assessed the effect of PEAME against *H. pylori* isolates, suggesting its potential in combination with metronidazole for eradication of this pathogen. The beneficial effect of PEAME may be attributed to its ability to enhance autophagy.

## 1. Introduction

Fruits and vegetables are considered a treasure of antioxidants and other constituents which synergistically promote health and lower the risk of many diseases including cancer, diabetes, neurodegenerative diseases, cardiovascular diseases, and microbial infections, among others [1,2]. Colored fruits and vegetables are rich in polyphenolic compounds and anthocyanins which are invaluable antioxidants with remarkable biological activities [2]. These constituents help fight many microbial and viral infections [2].

*Helicobacter pylori* is a commonly known Gram-negative, microaerobic spiral bacterium, which is considered among the most impactful chronic pathogens capable of causing human diseases. *H. pylori* infection causes chronic active gastritis, peptic ulcer, and gastric mucosa-associated lymphoma. Consequently, this pathogen has been classified by the WHO as a class I carcinogen which could promote gastric cancer [3]. Infection by *H. pylori* was reported in the literature in both children and adults [4,5]. The pathogenicity of *H. pylori* is attributed to certain virulence factors such as CagA, urease, IceA, and VacA, besides the bacterial adhesion and the maintenance factors. Moreover, the great genetic diversity of this bacterium relative to the host and environmental factors, as well as its ancestor strain, is controlled by some important virulence factors (CagA, IceA, and VacA) which collectively affect the disease severity [6].

Emergence of drug resistance represents a major challenge for eradicating this microbe despite the efficiency of the triple-management protocol followed in the empirical therapy of *H. pylori* infections [3,7]. In this respect, natural products exhibiting antimicrobial activities against such a pathogen might have a great potential for treating infections triggered by this microbe. 

Autophagy is a catabolic process activated in response to cellular stress, such as damaged organelles and accumulation of reactive oxygen species. Activated autophagy prevents excessive inflammatory response by breaking down the inflammatory mediators and preventing the activation of inflammasome [8]. Exposure of gastric epithelial cell culture to *H. pylori* reduces autophagy by preventing maturation of autophagosome. In addition, the autophagy-related gene expression is down-regulated in the gastric mucosa of patients with *H. pylori* infection, and this could provide a better habitat for *H. pylori* inside the gastric epithelial cells [9].

Pomegranate fruit (*Punica granatum* L., family Lythraceae) is rich in polyphenolic compounds and anthocyanins and has antioxidant, anticancer, anti-inflammatory, and antiulcer activities [10,11,12,13]. Intriguingly, punicalagin (a pomegranate constituent) and urolithin (a pomegranate metabolite), can induce autophagy in acute leukemia [14] and pancreatic β cells [15]. Pomegranate total peel extract demonstrated anti-*helicobacter* effect in vitro and in vivo [16]. However, the molecular mechanism of this effect is yet to be studied. The anti-*helicobacter* mechanism of anthocyanins was not assessed in vivo before.

Herein, we aimed to study the anti-inflammatory and autophagy-modulating mechanisms of pomegranate exocarp anthocyanin rich extract in the treatment of *H. pylori*-induced ulcers in vivo. Additionally, the combined effect of pomegranate extract and metronidazole was studied in vivo. We used the exocarp layer of the fruits’ peel and the chemical fingerprint of the extract was analyzed by UPLCMS/MS and ^1^H NMR.

## 2. Materials and Methods

This study includes the preparation of pomegranate exocarp anthocyanin methanol extract (PEAME), characterization of the polyphenolic content in PEAME, in vitro testing of the effect of PEAME on antibiotic resistant *H. pylori* clinical isolates obtained from gastric biopsies from patients suffering from dyspepsia. Additionally, an in vivo investigation of the antibacterial, anti-inflammatory, and autophagy mechanisms of PEAME alone or co-administered with metronidazole in *H. pylori* infected rats was carried out. Oxidative stress and antioxidant markers, such as malondialdehyde (MDA) and catalase activity, were measured. Gene expression analysis of tumor necrosis factor-alpha (TNF-α), *Beclin1*, and autophagy-related genes 5 and 12 (*ATG5* and *ATG12*, respectively) were performed. COX2 level was assessed using immunohistochemical staining. 

### 2.1. Plant Material, Extraction and Characterization

#### 2.1.1. Plant Material and Extraction

The fruits of *Punica granatum* L. Manfaloty cultivar were collected from a private garden in Manfalut City, Egypt. The identity was verified by the National Agriculture Research Center, Giza, Egypt. A voucher sample (PD-200-AR) was deposited at the department of Pharmacognosy, Faculty of Pharmacy, Tanta University.

The peel of the fruits was scraped by knifes to remove the mesocarp layer. The exocarp (5 kg) was cut into small pieces, air dried in the shade until completely dry and then kept in an oven at 40 °C for 1 h. The dried material was grinded into powder (2 kg). 

The powdered material was soaked with 70% methanol acidulated with HCl (0.5%) (20 mL solvent per each gram powdered material) at room temperature for a day using a magnetic stirrer then filtered. The process was repeated until exhaustion (after 7 days). The extract was evaporated under vacuum to leave a reddish-brown residue (100 gm).

#### 2.1.2. Characterization of the Contents of PEAME Extract

##### Determination of Total Polyphenolic Content

The total polyphenols content was measured using Folin–Ciocalteu method and gallic acid as a standard [17]. The contents were expressed as milligram (mg) equivalent to gallic acid/g extract.

##### Determination of Anthocyanin Content

The pH Differential method was followed according to Lee et al. [18]. The anthocyanin content was calculated as mg equivalent to cyanidin-3-*O*-glucoside/g extract.

##### Characterization of Pomegranate Exocarp Anthocyanin Methanol Extract (PEAME) by UPLC-PDA-MS/MS

The PEAME was analyzed by UPLC-PDA-MS/MS using a previously reported methodology [19,20]. Liquid chromatography system of A Nexera-i LC-2040 (Shimadzu, Kyoto, Japan) connected to a UPLC Shim-pack Velox C18 Column, 2.1 × 50 mm; 2.7 μm particle, was used. The solvent gradient and sample preparation were as reported [19,20]. An LC-2030/2040 PDA detector and an LC-MS 8045 triple quadrupole mass spectrometer equipped with electrospray ionization (ESI) source in negative mode (Shimadzu, Kyoto, Japan) were used for detection of compounds using the reported settings [19,20]. 

##### ^1^H NMR Fingerprint of PEAME

The ^1^H NMR analysis was performed using A Bruker Avance 400 spectrophotometer and DMSO-*d_6_* as a solvent at 400 MHz. The number of scans was 16 with a spectral width of 8012 Hz, an acquisition time of 4.089 s, and a relaxation delay of 1 s per scan. The FIDs were multiplied by an exponential weighting function corresponding to a line broadening of 0.3 Hz.

### 2.2. Ethical Statement

All procedures used in the current study were carried out based on the recommended Guide by the Faculty of Pharmacy Research Ethical Committee, Tanta University, Egypt (ethical approval code TP/RE/06/22P-0017). 

### 2.3. In Vitro Anti-H. pylori Effect of PEAME

#### 2.3.1. Collection of Gastric Biopsies and Isolation and Identification of the Test Pathogens

To obtain *H. pylori clinical isolates* for testing the biological effect of PEAME, 25 leftover samples of gastric mucosal biopsies were used to isolate *H. pylori*. As a diagnostic procedure, the samples were collected from the antrum using endoscopy obtained from children presented with dyspepsia (Age: 7–10 years), referred to the teaching hospitals of Tanta University. Biopsies were kindly provided by the staff members in the Department of Pediatrics Internal Medicine, Faculty of Medicine, Tanta University. Biopsies positive for *H. pylori* following urease test were transferred to sterile McCartney bottles containing Brain Heart Infusion (BHI) broth with 20% glycerol and 0.2 g/L cysteine. Bottles were transferred on ice to the microbiology laboratory within 60 min after collection for microbiological culture and analysis. 

Gastric biopsies were subjected to homogenization at 5000 rpm for 30 s to get a homogenous mixture of the gastric tissue in BHI broth. Each homogenized test sample was inoculated onto Mueller–Hinton agar supplemented with defibrinated 7% blood and antimicrobials (amphotericin B 3 mg/L, vancomycin 6 mg/L, polymyxin B 2500 IU/L). Plates were then incubated under microaerobic conditions at 37 °C for 3–7 days. Microscopy, as well as appropriate biochemical tests (urease, oxidase, and catalase), were performed for identification of *H. pylori*. Identification was carried out based on the Bergey’s Manual of Systematic Bacteriology [21].

#### 2.3.2. Susceptibility Test

To select the resistant strains for biological testing, susceptibility of *H. pylori* isolates to common therapeutic antimicrobials (clarithromycin, metronidazole, and amoxicillin) was performed according to the Clinical and Laboratory Standards Institute (CLSI, standard M7-A5, informational supplement M100-S10, 2000) [22]. Antimicrobials were tested in a concentration ranged between 0.016–256 µg/mL, then the percentage of test pathogens resistance to antimicrobials was calculated. The effect of pomegranate extract on *H. pylori* isolates was also investigated, and the minimum inhibitory concentration (MIC) and the minimum bactericidal concentration (MBC) were recorded. 

#### 2.3.3. Time-Kill Assay

To investigate the antimicrobial effect of PEAME in vitro, time course viability assay of the test pathogen was performed in the absence or presence of pomegranate extract at 1× MBC or 2× MBC as described by Ali et al. (2005). Samples were withdrawn at different time intervals (2, 4, 6, 8, 24, 48 h) to determine the viable count. Data were presented as the number of survivors (CFU/mL) against time (h). The experiment was repeated thrice, and the standard deviation (SD) was calculated.

### 2.4. Studying the Effect of PEAME on H. pylori Infection In Vivo

#### 2.4.1. Animals 

The study was performed in accordance with the guidelines for the care and use of laboratory animals approved by the Research Ethical Committee, Faculty of Pharmacy, Tanta University, Egypt. Forty-eight male Wistar rats, aged 3 months and weighed approximately 150–200 g, were purchased from the National Research Center (NRC, Cairo, Egypt). Rats were fed standard pellet chow (El-Nasr Chemical Company, Cairo, Egypt) and allowed free access to water. Animals were housed in normal temperature and normal dark/light cycle and maintained for one week for acclimatization. Forty rats were treated with streptomycin suspended in water (5 mg/mL) for three days before infection with *H. pylori*. After a day of fasting, rats were infected with *H. pylori* suspension by an oral inoculation of 1 mL 9 McFarland (2.7 × 10^9^ CFU) using gavage twice daily with 4 h interval, and for 8 sequential days. The control, non-infected group consisted of eight rats inoculated with sterile phosphate buffered saline (PBS) [16]. Infected rats were divided equally into five groups as follows: *H. pylori* infected control (PC) group (rats were given the vehicle (distilled water) orally and daily for 3 weeks); pomegranate prophylactic (Pom. Proph.) group (rats were given PEAME orally at a dose of 100 mg/kg daily three days before *H. pylori* infection and continued for 2 weeks after infection) [16]; pomegranate treatment (Pomt. Treat.) group (infected rats were given PEAME at a dose of 100 mg/kg orally and daily after 7 days of the last *H. pylori* inoculation and the treatment is continued for one week) [16]; metronidazole treatment (Met. Treat.) group (infected rats were given metronidazole at a dose of 10 mg/kg orally and daily after 7 days of the last *H. pylori* inoculation and the treatment continued for one week) [23]; and pomegranate and metronidazole co-treated (Pom + Met. Treat.) group (infected rats were given PEAME and metronidazole at a dose of 100 mg/kg and 10 mg/kg, respectively, orally and daily after 7 days of the last *H. pylori* inoculation and the treatment continued for one week).

At the end of the experiment, the weight of rats was determined, and the rats were sacrificed. The stomach was removed and washed with isotonic saline. Each stomach was cut into small portions. One portion was used for preparation of tissue homogenate in isotonic saline (10% *w*/*v*). The homogenates were then centrifuged at 3000 rpm for 15 min and the supernatant was used for culture, as described above, following similar growth conditions. Another portion was fixed in formalin (10%) for histopathological examination, while the finally remaining portion was kept in −80 °C for RT-qPCR and biochemical estimations (determination of oxidative stress and antioxidant markers). 

#### 2.4.2. Determination of Oxidative Stress and Antioxidant Markers

Stomach tissues were homogenized in 0.1 mmol/L phosphate buffer (pH 7.4). The homogenates (10% *w*/*v*) were then centrifuged at 4000 rpm for 15 min and the supernatant was used for the biochemical estimations of lipid peroxidation markers and catalase activity [24].

##### Markers of Lipid Peroxidation

The degree of stomach tissue lipid peroxidation was assayed spectrophotometry using lipid peroxide (Malondialdehyde) kit Biodiagnostic^®^, Cairo, Egypt. According to the kit instructions, a pink color is produced from the reaction of thiobarbituric acid with malondialdehyde (MDA) which absorbs light at 534 nm [25].

##### Catalase Activity

Catalase activity was assessed following a previously published method [26], wherein the breakdown of H_2_O_2_ was measured. A mixture of 0.05 mL of stomach homogenate, 3 mL of H_2_O_2_ and phosphate buffer was prepared. The change in the absorbance of the mixture was measured every 30 s for two min interval at 240 nm using a spectrophotometer (Analytik, Jena, Italy). 

#### 2.4.3. Assessing the Expression of TNF-α and Autophagy Genes Using Quantitative Real Time-Polymerase Chain Reaction (RT-qPCR)

To investigate the anti-inflammatory and autophagy mechanisms, gene expression analysis of tumor necrosis factor-alpha (TNF-α), *Beclin1*, and autophagy-related genes 5 and 12 (*ATG5* and *ATG12*, respectively) was performed using RT-qPCR. Total RNA was extracted from the stomach tissue using RNeasy^®^ Mini kit (Qiagen^®^, Hilden, Germany). The concentration of the extracted RNA was measured using Nanodrop (Denovix^®^, Wilmington, DE, USA). An amount of 1 μg of the extracted RNA was used for the synthesis of cDNA by QuantiTect^®^ reverse transcription kit (Two Step RT-PCR Kit, Qiagen Co., Hilden, Germany). QuantiTect SYBR Green I PCR (Qiagen^®^, Hilden, Germany) was used for RT-PCR. Primers were purchased from Biosearch Technologies^®^ (Novato, CA, USA). *Beclin1* (NM-001034117.1); forward (5′-GCCTCTGAAACTGGACACG-3′), reverse (5′-CCTCTTCCTCCTGGCTCTCT-3′), *ATG5* (NM_001014250.1); forward (5′-CACTGGGACTTCTGCTCCTG-3′), reverse (5′-TTCTTCAACCAAGCCAAACC-3′). *ATG12* (NM_001038495.1); forward (5′-AAACGAAGAAATGGGCTGTG-3′), and reverse (5′-GAAGGGGCAAAGGACTGATT-3′) [27], *TNF*-*α* (NM_012675.3); forward (5′-ACT GAA CTT CGG GGT GAT TG-3′), reverse (5′-GCT TGG TGG TTT GCT ACG AC-3′) and *GAPDH* (NM-017008.4); forward (5′-CGC TCT CTG CTC CTC CTG TT-3′), reverse (5′-CCA TGG TGT CTG AGC GAT GT-3′) [28]. The relative gene expression was normalized to GAPDH as an endogenous control using 2^−ΔΔCT^ method [29].

#### 2.4.4. Histopathological Evaluation 

At the end of our experiment, the stomach sections of four rats were chosen randomly from each group to evaluate the histopathological changes in the gastric mucosa of rats following the treatment. The stomach tissue was fixed in 10% neutral buffered formalin overnight. Each sample was then embedded in paraffin and sections (4 μm thick) were cut and stained with hematoxylin-eosin (HE) for morphological analysis. The inflammation score was used for recording and grading the histopathological findings as follows: 0, no infiltration of leukocytes; 1, mild infiltration of leukocytes; 2, moderate infiltration of leukocytes; and 3, severe infiltration of leukocytes. Gastritis was classified according to Sydney System. A modified score (0–3) was assigned to metaplasia and mononuclear cells infiltration according to Sozzi et al. [30].

#### 2.4.5. Immunohistochemical Staining of Cyclooxygenase 2 (COX2)

To evaluate the effect of PEAME on COX2 level, immunohistochemical (IHC) staining of COX2 in paraffin-embedded stomach sections was performed using COX2 kit (Sigma Aldrich^®^, Burlington, VT, USA). Stomach tissues fixed in 10% neutral formalin and B-5 fixative were used. Tissue sections were cut at 4 μm. The sections were deparaffinized and hydrated. Endogenous peroxidase was quenched with two drops of 3% H_2_O_2_ for 5 min. Then, the sections were incubated with a blocking reagent for ten min. The primary antibody (Rabbit anti-cyclooxygenase 2 in buffered saline, dilution 1:200) or negative control was applied and incubated for 1 h. Then, biotinylated secondary antibody (Goat anti-rabbit IgG in buffered saline, dilution 1:200) was applied and incubated for 20 min. Peroxidase reagent was applied and incubated for 20 min. A substrate reagent and 3-amino-9-ethylcarbazole in *N*,*N*-dimethylformamide chromogen were applied for 10 min. IHC counterstained with Mayer’s hematoxylin. Staining showed nuclei in blue, while the cytoplasm of positive cells in rose-red to brownish red. The intensity of the positive IHC expression was blindly scored in all immunostained slides from 0–3 as 0 when negative, 1 when mild, 2 when moderate, and 3 when strong [31].

#### 2.4.6. Bacteriological Analysis

To evaluate the antibacterial effect of PEAME in vivo, collected gastric tissues from each test group were weighed then subjected to homogenization in about 10 mL normal saline. Plating of serially 10-fold diluted homogenates was carried out using blood supplemented Mueller–Hinton agar. Results were expressed as mean values of log_10_ CFU per gram of gastric tissue and the standard deviation (SD) was determined. The bactericidal effect was defined as equal to or more than 3 log_10_ reductions compared to the initial count calculated at zero time. For synergy, it was defined as equal to or more than 2 log_10_ reductions in case of drug combinations, in comparison to the most active single drug. Experiments were performed in triplicate [16,32].

#### 2.4.7. Statistical Analysis

Analysis of data was performed with the statistical package for social science (SPSS) software version 22 [33]. Data are presented as mean ± SD or SE. Statistical comparison among groups was performed using one-way analysis of variance (ANOVA) followed by post-hoc analysis. Statistical significance was set at *p* < 0.05. 

## 3. Results

### 3.1. Characterization of Polyphenolics in PEAME

**Figure 1:**
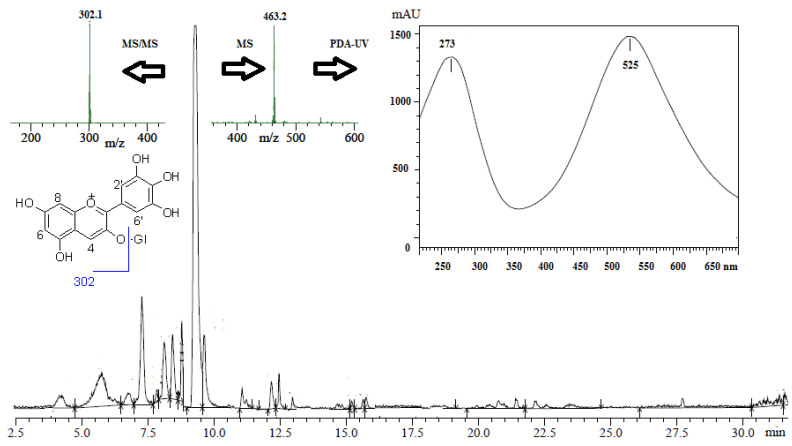
Total ion chromatogram of PEAME analyzed by UPLC-PDA-MS/MS in negative ion mode, showing the *m*/*z* 463 at retention time 8.12 min, its PDA-UV absorbance at λ_max_ at 273 and 525 nm, and its MS/MS fragmentation to *m*/*z* 302. The structure of delphinidin-3-*O*-glucoside is shown.

In this study, the total polyphenolic content of PEAME was 537 mg equivalent to gallic acid per gram of the dry PEAME extract which indicated the high content of polyphenolic compounds in the exocarp extract. The anthocyanin content was calculated as 380 mg equivalent to cyanidine-3-*O*-glucoside per gram of the dry PEAME which implied that the extract is rich in anthocyanins. The UPLC-PDA-MS/MS analysis in a negative ion mode revealed 10 peaks (Figure 1). Compounds were identified (Table 1) based on the retention times, mass and MS/MS data compared to the published data for pomegranate [34,35,36]. The anthocyanins identified are delphinidin-3-*O*-glucoside and cyanidine-3-*O*-glucoside. Moreover, the tannins related compounds pedunculagin I and II, punicalagin, punigluconin, valoneic acid dilactone, ellagic acid-*O*-pentoside, ellagic acid, and hexahydroxy diphenoyl glucoside (HHDP-glucoside) were detected. Based on the peak area, the major anthocyanin identified was delphinidin-3-*O*-glucoside with an [M-H]^−^ molecular ion at *m*/*z* 463, which produced an MS/MS fragment of [M-162]^−^ at *m*/*z* 302 for delphinidin aglycone after loss of glucosyl moiety (Figure 1). The PDA-UV absorbance spectrum of the *m*/*z* 463 indicated λ_max_ at 273 and 525 nm which is characteristic for delphinidin-3-*O*-glucoside (Figure 1).

**Figure 2:**
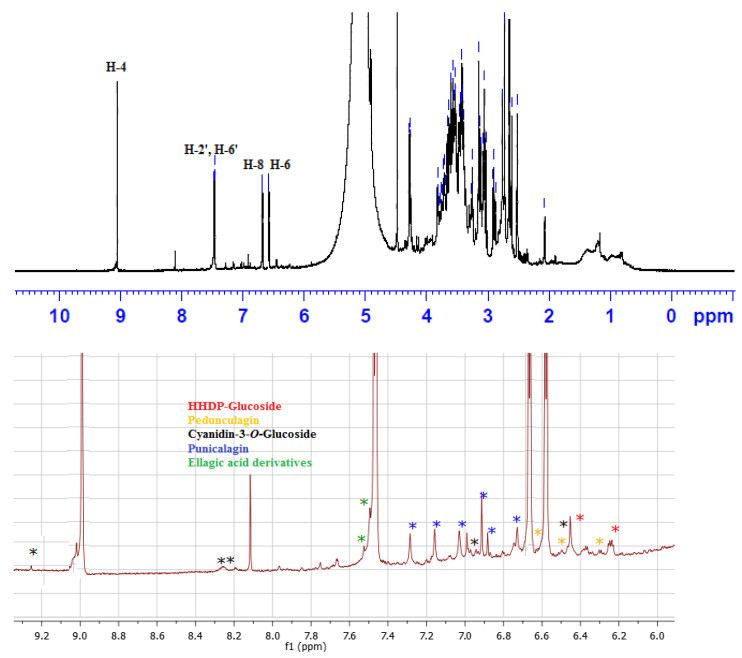
^1^H NMR spectrum of PEAME showing the characteristic peaks for delphinidin-3-*O*-glucoside (**Top**), the expansion to show the characteristic peaks in the aromatic region (6.00–9.30 ppm) for the other compounds (**bottom**).

The ^1^H NMR analysis of the extract (Figure 2) indicated that the characteristic signals for delphenidin-3-*O*-glucoside were predominant compared to the published literature [37]. The signals at δ_H_ 6.5 and 6.7 were assigned to H-6 and H-8, respectively, while the doublet at δ_H_ 7.46 integrating for two protons was assigned to H-2′ and H-6′. The singlet at δ_H_ 9.1 was ascribed to H-4. Accordingly, delphinidin-3-*O*-glucoside was considered the major compound in the extract. Signals for cyanidin-3-*O*-glucoside appeared at δ_H_ 6.44, 6.93, 8.21, 8.26, and 9.25 which are consistent with the literature [37]. Additionally, signals at δ_H_ 6.25 and 6.43 were assigned to H-3′ and H-3′’ of HHDP-glucoside [38]. Signals at 7.50 and 7.52 were ascribed to H-5,5′ of ellagic acid derivatives [39]. Signals at δ_H_ 6.72, 6.88, 6.90, 6.97, 7.05, 7.12, 7.21, and 7.26 were attributed to the aromatic protons of α and β punicalagin compared to the literature [40]. Signals at δ_H_ 6.30, 6.31, 6.50, 6.54, 6.59, 6.60, 6.63, and 6.64 were consistent with pedunculagin [41].

### 3.2. In Vitro Anti-H. pylori Effect of PEAME

**Figure 3:**
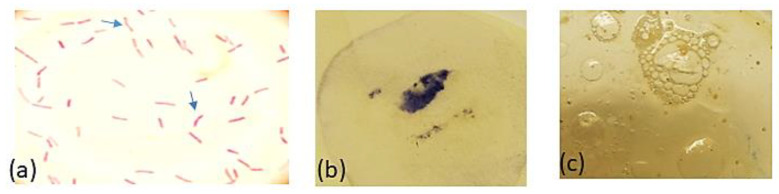
(**a**) Gram-stained *H. pylori* cells showing slightly curved, red-colored rods (arrows) and some are straight rods with blunt ends; (**b**) positive oxidase test; and (**c**) positive catalase test showing effervescence after subjection to hydrogen peroxide solution.

The *H. pylori* resistant strains were isolated for testing the antimicrobial effect of PEAME. A total of 17 (68%) *H. pylori* isolates were recovered out of 25 antral gastric biopsies obtained from dyspeptic children through esophagogastroduodenoscopy. The identification of *H. pylori* isolates was based on the morphology of the colony which appeared as translucent dome-shaped and small in size (1 mm or less in diameter). Additionally, upon performing Gram-staining technique and light microscopy examination, it showed a Gram-negative reaction in the form of slightly curved to spiral red-colored rods and some were straight rods with blunt ends as observed in Figure 3. Furthermore, *H. pylori* isolates showed positive results for all the three enzymatic tests (catalase, oxidase, and urease). Further identification parameters are summarized in Table 2.

**Figure 4:**
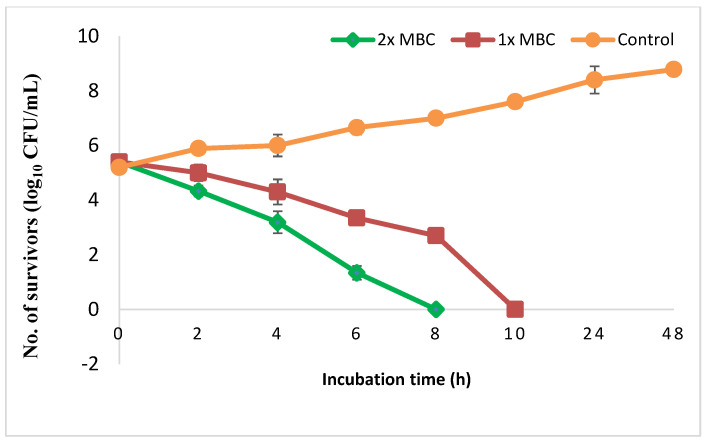
Time-kill assay of the test pathogen was performed in the absence or presence of pomegranate extract at 1× MBC or 2× MBC.

Susceptibility test of *H. pylori* isolates to different therapeutic antimicrobials revealed that metronidazole was inactive against 53% of the isolates followed by clarithromycin (41%) as recorded in Table 3. The resistant isolates were selected and subjected to PEAME that showed activity against all of them recording MIC and MBC values of 128 and 256 µg/mL, respectively. Figure 4 presents the time-kill assay of *H. pylori* by pomegranate peel extract. Treatment of the test pathogen by 1× MBC resulted in approximately 2-log_10_ reductions in the viable count after 6 h of incubation. This decrease in CFU continued until reached up to 5 log_10_ reductions at 10 h compared to the initial count recorded at zero time indicating bactericidal effect. Moreover, no viable count was recorded at 8 h when *H. pylori* was exposed to 2× MBC.

### 3.3. In Vivo Anti-H. pylori Effect of PEAME

Following the in vivo infection of rats by *H. pylori* and treatment with PEAME alone or with metronidazole as detailed in the experimental section, some criteria were assessed including the effect on the weight of the rats, colonization of *H. pylori* in the stomach tissue of rats, lipid peroxidation markers, gene expression of TNF-α and autophagy genes in the stomach tissue of rats, histopathological investigations and assessing the level of COX2 in the stomach tissues.

#### 3.3.1. Effect on the Weight of the Rats

**Figure 5:**
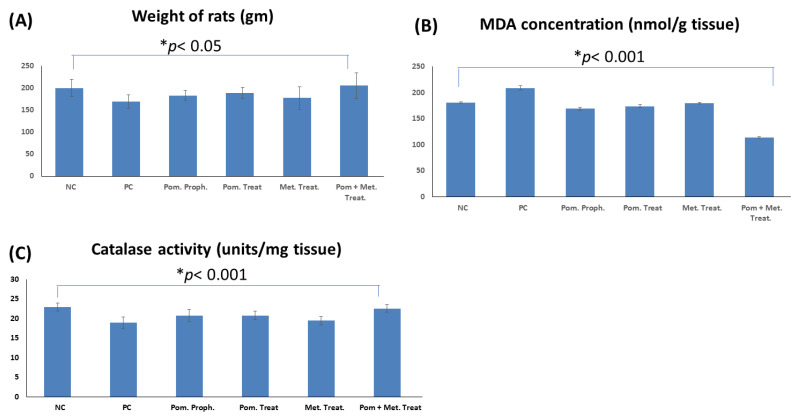
Effect of *H. pylori* infection, pomegranate extract (PEAME), and metronidazole on the weight of rats (**A**), malodialdehde (MDA) concentration in stomach tissue (**B**), and catalase activity in stomach tissue (**C**). Data are presented as mean ± SD, *n* = 8, significance level *p* < 0.05. NC: negative control (not infected), PC: positive control (infected with *H. pylori*), Pom proph.: PEAME prophylactic group, Pom treat.: PEAME treated group, Met. Treat.: metronidazole treated group, Pom + Met treat.: rats co-treated with PEAME and metronidazole.

As the infection with *H. pylori* decreases the appetite and, consequently, the weight, at the end of the experiment the weight of rats in different groups was measured. Our results showed that the weight of rats in *H. pylori* infected (positive control) group was significantly lower than the weight of the animals in the normal control group (*p* < 0.05). Furthermore, the weight of the animals co-treated with PEAME and metronidazole was significantly higher than the weight of the animals in the positive control group (*p* < 0.01) and metronidazole only treated group (*p* < 0.05) (Figure 5A).

#### 3.3.2. Effect on the Colonization of *H. pylori* in the Stomach of the Infected Rats

Table 4 presents the reduction in the viable count of *H. pylori* colony forming units (CFU) following subjection of infected rats to different treatments. It was observed that using metronidazole alone as a treatment decreased the number of survivors to 3.54, but it was a non-significant reduction compared to the positive control (4.11). Similar results were recorded for the groups exposed to pomegranate either as a treatment or prophylactic as shown in Table 4. It is worth mentioning that combination of pomegranate with metronidazole dramatically reduced the viable count of *H. pylori* by approximately 3 log reductions as recorded in Table 4.

#### 3.3.3. Effect on the Lipid Peroxidation of the Stomach Tissue in the Rats Infected with *H. pylori*

As *H. pylori* infection causes an increase in the lipid peroxidation as evidenced by an increase in the concentration of MDA, the effect of PEAME on lipid peroxidation was evaluated. In this study MDA concentration was measured in the stomach tissue homogenate as a marker of lipid peroxidation. MDA concentration in the stomach of rats infected with *H. pylori* (positive control) was significantly higher than the uninfected rats in the negative control group (*p* < 0.001). Prophylactic administration of PEAME as well as treatment with PEAME or metronidazole or combined administration of PEAME and metronidazole significantly decreased MDA concentration in the treated rats compared to the untreated rats in the positive control group (*p* < 0.001). More interestingly, the infected rats co-treated with PEAME and metronidazole showed a significant decrease in MDA concentration compared to the other studied groups (*p* < 0.001) (Figure 5B).

#### 3.3.4. Effect on the Catalase Activity in the Stomach Tissue of the Rats Infected with *H. pylori*

As the infection with *H. pylori* causes a decrease in catalase activity, the effect of PEAME on catalase activity was investigated. The current study showed that the infection with *H. pylori* significantly decreased catalase activity (*p* < 0.001). Prophylactic administration of PEAME as well as treatment with PEAME alone or combined treatment with PEAME and metronidazole induced a significant increase in catalase activity in the stomach tissue of the infected rats (*p* < 0.001). In addition, the catalase activity in the stomach of the infected rats co-treated with PEAME and metronidazole was significantly higher than the catalase activity in the stomach of the infected rats in pomegranate prophylactic group (*p* < 0.05), pomegranate treated group (*p* < 0.05), and metronidazole treated group (*p* < 0.01) (Figure 5C). 

#### 3.3.5. Effect on the Gene Expression of TNF-α in the Stomach Tissue of the Rats Infected with *H. pylori*

**Figure 6:**
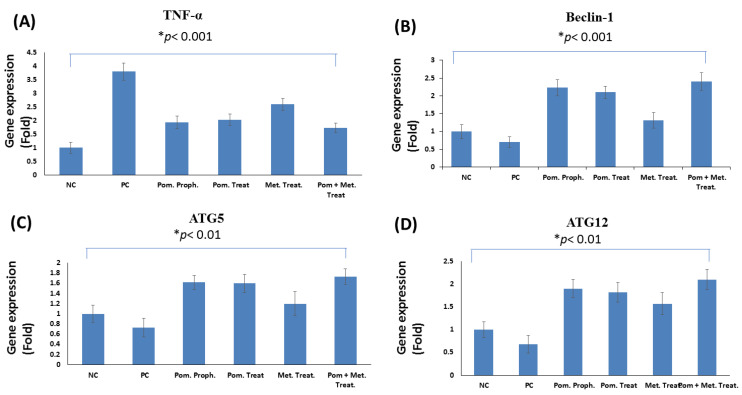
Effect of *H. pylori* infection, pomegranate extract (PEAME) and metronidazole on the mRNA expression levels of (**A**) tumor necrosis factor-alpha (*TNF*-*α*), (**B**) autophagy-related gene 5 (*ATG5*), (**C**) autophagy-related gene 12 (*ATG12*), and (**D**) *Beclin1* in the stomach tissues of rats. Data are presented as mean ± SE, *n* = 3, significance level *p* < 0.05. NC: negative control (not infected), PC: positive control (infected with *H. pylori*), Pom proph.: PEAME prophylactic group, Pom treat.: PEAME treated group, Met. treat.: metronidazole treated group, Pom + Met treat.: rats co-treated with PEAME and metronidazole.

Infection with *H. pylori* causes inflammation of the gastric mucosa; therefore, in the present study, *TNF-α* gene expression was measured in the stomach tissue as a marker of inflammation. *TNF-α* gene expression in the stomach of the rats infected with *H. pylori* (positive control) was significantly higher than the uninfected rats in the negative control group (3.8-fold), *p* < 0.01. Prophylactic administration of PEAME as well as treatment with PEAME or metronidazole or combined administration of PEAME and metronidazole in the infected rats significantly decreased TNF-α gene expression (1.94, 2.03, 2.60, and 1.75-fold, respectively) compared to the untreated rats in the positive control group (3.80-fold), *p* < 0.01, *p* < 0.01, *p* < 0.05, *p* < 0.001, respectively (Figure 6A).

#### 3.3.6. Effect on the Expression of Autophagy Genes in the Stomach Tissue of the Rats Infected with *H. pylori*

For exploring the potential autophagy mechanism of PEAME, the expression of the key autophagy genes was measured. Our RT-qPCR results showed that infection with *H. pylori* decreased the expression of the autophagy genes (*Beclin1*, *ATG5*, and *ATG12* expression decreased by 0.71-, 0.73-, and 0.68-fold, respectively) compared to the negative control group (1-fold). Prophylactic administration of PEAME as well as treatment with PEAME alone or combined treatment with PEAME and metronidazole induced a significant increase in the expression of autophagy genes (*Beclin1*, *ATG5*, and *ATG12*) in the stomach tissue of the infected rats compared to the stomach of the untreated rats in the positive control group (*p* < 0.05). Out results showed that the combined treatment with PEAME and metronidazole significantly increased the gene expression of the autophagy genes (*Beclin1*, *ATG5*, and *ATG12* expression increased by 2.4-, 1.78-, and 2.1-fold, respectively) in the stomach tissue of the infected rats compared to the stomach of rats in the positive control group (0.71-, 0.73-, and 0.68-fold, respectively, *p* < 0.01) (Figure 6B–D). 

#### 3.3.7. Histopathological Investigations

**Figure 7:**
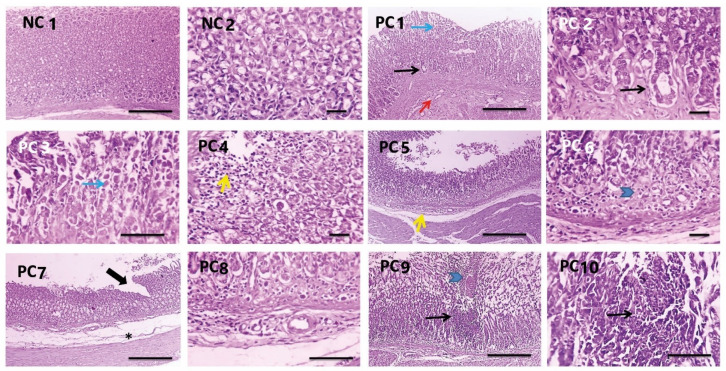
Microscopic pictures of H & E-stained glandular gastric mucosa showing normal gastric glands in the control normal group (NC1,2). Meanwhile, glandular gastric mucosa from the rats infected with *H. pylori* (PC1–10) showing metaplasia (black arrows) apoptosis (blue arrows), necrosis (blue arrowheads), focal ulceration (thick arrow) with submucosal congestion (red arrows), edema (*), and mononuclear cells infiltration (yellow arrows). Low magnification X: 100 bar 100 and high magnification X: 400 bar 50.

Histopathological changes in the rat gastric mucosa following infection with *H. pylori* and the treatment with PEAME were investigated to assess the degree of inflammation and tissue damage. Histopathological examination revealed that the glandular gastric mucosa from the rats infected with *H. pylori* showed metaplasia, apoptosis, necrosis, focal ulceration with submucosal congestion, edema, and mononuclear cells infiltration. Meanwhile, the glandular gastric mucosa of the rats in the normal control group showed normal gastric glands (Figure 7).

**Figure 8:**
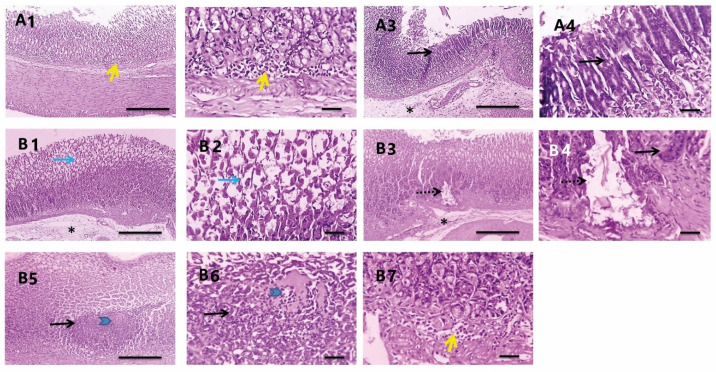
Microscopic pictures of H&E-stained glandular gastric mucosa from *H. pylori* infected rats treated with PEAME (**A1**–**A4**) showed metaplasia (black arrows) with low mononuclear cell infiltration (yellow arrows) and submucosal edema (*) in some sections. Meanwhile, glandular gastric mucosa from *H. pylori* infected rats treated with metronidazole (**B1**–**B7**) showed apoptosis (blue arrows), adenoma (dashed black arrows) with metaplasia (black arrows), necrosis (blue arrowheads), mononuclear cells infiltration (yellow arrows) and submucosal edema (*). Low magnification X: 100 bar 100 and high magnification X: 400 bar 50.

The glandular gastric mucosa from *H. pylori* infected rats treated with PEAME showed metaplasia with few mononuclear cells’ infiltration and submucosal edema in some sections (Figure 8A). Meanwhile, the glandular gastric mucosa from *H. pylori* infected rats treated with metronidazole showed apoptosis, metaplasia, necrosis, mononuclear cells infiltration, and submucosal edema (Figure 8B).

**Figure 9:**
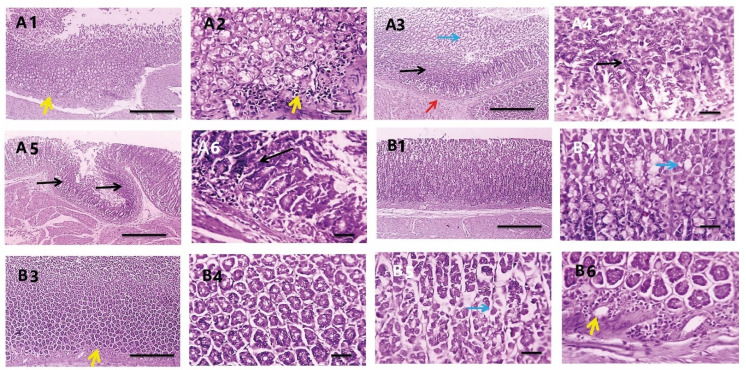
Microscopic pictures of H&E-stained glandular gastric mucosa from *H. pylori* infected rats taking prophylactic PEAME (**A1**–**A6**) showed metaplasia (black arrows), submucosal congestion (red arrows), apoptosis (blue arrows) with low mononuclear cell infiltration (yellow arrows). Meanwhile, the glandular gastric mucosa from *H. pylori* infected rats co-treated with PEAME and metronidazole (**B1**–**B6**) showed improved histology with mild apoptosis (blue arrows). Low magnification X: 100 bar 100 and high magnification X: 400 bar 50.

In addition, the glandular gastric mucosa from *H. pylori* infected rats taking prophylactic PEAME showed metaplasia, apoptosis with few mononuclear cells’ infiltration (Figure 9A). More interestingly, the glandular gastric mucosa from infected rats co-treated with PEAME and metronidazole showed less frequent and milder apoptosis (Figure 9B, Table 5).

#### 3.3.8. Effect on the COX2 Level 

**Figure 10:**
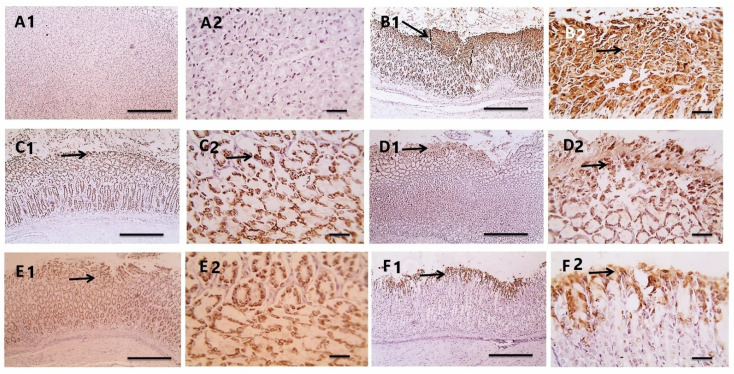
Microscopic pictures of H&E-stained glandular gastric mucosa from *H. pylori* infected rats treated with PEAME (**A1**,**A2**) showing metaplasia (black arrows) with few mononuclear cells’ infiltration and submucosal edema in some sections. Meanwhile, the glandular gastric mucosa from *H. pylori* infected rats treated with metronidazole (**B1**,**B2**) showed apoptosis, adenoma with metaplasia (black arrows), necrosis, mononuclear cells infiltration, and submucosal edema. (**C1**,**C2**) the glandular gastric mucosa from *H. pylori* infected rats taking PEAME prophylactic. (**D1**,**D2**) the glandular gastric mucosa from *H. pylori* infected rats taking PEAME treatment. (**E1**,**E2**) the glandular gastric mucosa from *H. pylori* infected rats taking metronidazole. (**F1**,**F2**) the glandular gastric mucosa from *H. pylori* infected rats taking PEAME and metronidazole. Low magnification X: 100 bar 100 and high magnification X: 400 bar 50.

To further confirm inflammation, we performed immunostaining of COX2. Immunostained gastric mucosa against COX2 showed negative expression in the normal control group (Figure 10A). Meanwhile, the gastric mucosa from the rats infected with *H. pylori* showed strong diffuse positive brown expression and mainly in foveolar epithelium (Figure 10B). A reduction of the positive brown expression appeared in the glandular gastric mucosa from *H. pylori* infected rats taking PEAME prophylactic (Figure 10C) or PEAME treatment (Figure 10D) or metronidazole treatment (Figure 10E) as diffusely in the whole mucosa and slightly higher in foveolar epithelium. Marked reduction of the positive brown expression appeared in gastric mucosa from the infected rats co-treated with PEAME and metronidazole that was noticed only in foveolar epithelium (Figure 10F, Table 5).

## 4. Discussion

*H. pylori* is a major causative agent of peptic ulcer in humans, whether adults or children [4,5]. Despite the availability of treatment, the resistance to antibiotics in many patients leads to therapeutic failure [3]. A previous study involving Egyptian schoolchildren has reported a high prevalence (72.38%) of *H. pylori* infection [42] with a higher infection rate in the Upper Egypt (96.7% in Suhag) compared to Cairo and Giza (61.9%). Therefore, we isolated this pathogen from children patients suffering from dyspepsia. Comparable results were reported in the current study where *H. pylori* isolates were recovered in 68% of antral gastric biopsies obtained from dyspeptic children. The findings of many studies revealed that contaminated water is likely the main source of transmission of *H. pylori* infection [43]. *H. pylori* was detected, using PCR, in water samples from certain geographical locations in Egypt, particularly Abu El Matamir-Beheira, and Sidi Bishr-Alexandria. Therefore, geographical location, socio-economic status, and sociodemographic variables could impact the infection prevalence. 

In the present work, susceptibility of *H. pylori* isolates to different therapeutic antimicrobials revealed that metronidazole was ineffective in 53% of the treated isolates followed by clarithromycin (in 41% of the treated isolates). Similarly, Otaga et al. [44] reported that *H. pylori* increasingly develop antimicrobial resistance, particularly to clarithromycin and metronidazole). Hence, under empirical therapy, there is a risk of eradication failure which is usually associated with development of secondary resistance. Accordingly, searching for either synthetic or natural alternatives is essential to reduce the development of antibiotic resistance in *H. pylori* pathogen. Anthocyanins are natural antioxidants [45], potentially effective against *H. pylori* as they inhibit adhesion of *H. pylori* to the human gastric mucus in vitro [46,47]. Cyanidin-3-*O*-glucoside, a major anthocyanin in many plants including pomegranate, was reported to suppress the transcription of secA in *H. pylori* [44]. SecA is a protein responsible for the extracellular secretion of the toxins CagA and VacA, which, in turn, are involved in the pathogenesis of *H. pylori* [48]. Thus, we expected that pomegranate extract rich in anthocyanins would exert a promising effect to eradicate the pathogen. Exocarp of pomegranate peel is the outer colored layer and its characteristic color is due to anthocyanins [45]. Many solvents were used in previous studies for the extraction of anthocyanins, of which the extraction with acidulated aqueous methanol is the classical method and was recommended for higher yields [49,50]. Thus, 70% methanol acidified with HCl was selected in our study for preparing PEAME with high yield of anthocyanins from the pomegranate exocarp. In many pomegranate cultivars, cyanidine-3,5-di-*O*-glucoside and cyanidine-3-*O*-glucoside are the major anthocyanins [51]. However, in some others 18elphinidin-3-*O*-glucoside was the major one [52]. Herein, delphenidine-3-*O*-glucoside was the major anthocyanin in the exocarp of the Manfaloti cultivar based on the intensity of the ^1^H NMR peaks. In our study, the resistant isolates of *H. pylori* were selected and subjected to PEAME that showed activity against all of them recording MIC and MBC values of 128 and 256 µg/mL, respectively. Moreover, data of time-kill assay of *H. pylori* by PEAME displayed bactericidal effect where no viable count was recorded at 8 h when *H. pylori* was exposed to 2x MBC.

It has been reported that infection with *H. pylori* increases oxidative stress in the stomach tissue [53]. Our results showed that infection of the rats with *H. pylori* caused a significant increase in lipid peroxidation, as revealed by an increased MDA concentration (*p* < 0.001), and a significant decrease in catalase activity (*p* < 0.001) in the stomach tissue. 

Furthermore, PEAME alone or combined with metronidazole significantly reduced the oxidative stress via a reduction of MDA level and an increase in the antioxidant activity of catalase in the stomach tissue of rats infected with *H. pylori* (*p* < 0.001). These results were supported by Sayed et al. [54], who showed that pomegranate peel extract significantly elevates catalase activity, reduces MDA, and has strong antioxidant properties against induced hepatotoxicity in rats.

Moreover, this study showed that the treatment with metronidazole significantly reduced MDA level in the stomach tissue of rats infected with *H. pylori* (*p* < 0.001). Current results are in agreement with the study of Szentmihályi et al. [55] which revealed that metronidazole inhibits the induced lipid peroxidation in vitro and it has non-direct antioxidant properties.

Tumor necrosis factor-alpha (TNF-α), is a pro-inflammatory cytokine produced during inflammation. TNF-α participates in vasodilatation and edema formation [56]. The combined treatment with PEAME and metronidazole significantly ameliorated the increase in the gene expression of *TNF-α* in the stomach tissue of rats infected with *H. pylori*. These results were supported by histopathological evaluation which showed that the combined treatment with PEAME and metronidazole ameliorated inflammation and tissue damage caused by *H. pylori* infection in rats’ stomachs.

The inducible enzyme COX2 catalyzes the conversion of arachidonic acid to prostaglandins and plays a vital role in the inflammatory process [57]. Our immunohistochemical results showed that the combined treatment with PEAME and metronidazole alleviated the increase in the number of COX2 positive cells caused by *H. pylori* infection in rat stomachs. The present study was in-line with Sayed et al. [54] who found that PEAME normalizes COX2 and has anti-inflammatory activity that protects against hepatotoxicity in rats. 

Autophagy, a highly regulated pathway, can degrade damaged and aging organelles for recycling, thus playing a vital role in maintaining the stability of the internal environment [8]. The hallmark of autophagy is the formation of double-membrane vesicles called autophagosomes, which is regulated by proteins encoded by autophagy-related genes (ATGs) [58]. 

In the current study we determined the gene expression of autophagy genes (*ATG5*, *ATG12*, and *Beclin1*). Our RT-qPCR results showed that the infection with *H. pylori* decreased the expression of autophagy genes compared to the negative control group. Prophylactic administration of PEAME as well as treatment with PEAME alone or combined with metronidazole induced a significant increase in the expression of autophagy genes in the stomach tissue of the infected rats compared to the stomach of the untreated rats in the positive control group (*p* < 0.05). 

In support of our results, Tanaka et al. [9] revealed that, *H. pylori* lysate can induce autophagy in gastric epithelial cells in vitro. However, continuous prolonged exposure to *H. pylori* decreases autophagy by inhibiting maturation of the autophagosome. Additionally, Subkorn et al. [14] indicated that punicalagin (a pomegranate compound) can induce autophagy in acute leukemia. Moreover, another study by Zhang et al. [15] showed that, urolithin A (a pomegranate metabolite) activates autophagy in pancreatic β-cells.

In our study, the observed results could be attributed to the chemicals identified in the extract, namely, the anthocyanins delphinidin-3-*O*-glucoside, cyanidine-3-*O*-glucoside, the tannins, and related compounds. 

## 5. Conclusions

Combined treatment with PEAME and metronidazole significantly inhibited the colonization of *H. pylori* and decreased the associated inflammation and oxidative stress via enhancing autophagy.

## Figures and Tables

**Table 1:** UPLC-MS/MS identification of the compounds in PEAME. R_t_: retention time. Anthocyanins are highlighted in green. (*) denotes a molecular ion of [2M-H]^−^.

NO.	R_t_ min	[M-H]^−^ *m*/*z*	MS^2^ Ions *m*/*z*	Identification
1	4.25	783	481, 301, 275	Pedunculagin I
2	5.76 7.27	541 * 1083	541, 302 1083, 781, 603, 601, 575, 541, 302	Punicalagin (α, β)
3	6.75	783	765, 481, 301, 275	Pedunculagin II
4	7.84	801	649, 348, 347, 301	Punigluconin
5	8.12	463	302	Delphenidin-3-*O*-glucoside
6	8.43	469	425, 301, 169, 125	Valoneic acid dilactone
7	8.87	447	345, 259, 219, 160, 113	Cyanidin-3-*O*-glucoside
8	9.29	433	301	Ellagic acid-*O*-pentoside
9	11.06	301	301, 229, 185	Ellagic acid
10	12.50	481	301	HHDP-glucoside

**Table 2:** Morphological and biochemical characteristics detected among *H. pylori* isolates.

Test Parameter	Observation
Colony morphology	Round, small, translucent, 2–3 mm
Bacterial cell shape	Spiral, helical or curved with blunt ends
Gram staining reaction	Negative
Urease	Positive
Catalase	Positive
oxidase	Positive
Motility	Positive
Nitrate reduction	Negative
Glycine utilization	Negative
Growth on blood agar	Positive

**Table 3:** Susceptibility of *H. pylori* isolates to common therapeutic antimicrobials.

Antimicrobial Agents	Resistance (%)	MIC Range (µg/mL) *
Metronidazole	53	0.5–64
Clarithromycin	41	0.5–16
Amoxicillin	38	0.25–64

* Strains were considered resistant if MIC breakpoints ≥1 µg/mL for all except metronidazole (≥8 µg/mL).

**Table 4:** The viable count of *H. pylori* colony forming units (CFU) following subjection of infected rats to different treatments.

Group	Test Groups	Log No. of Survivors ± SD
1	Negative control	0 ± 0 *
2	Positive control	4.11 ± 0.22
3	Pomegranate Prophylaxis	3.71 ± 0.23
4	Treatment with pomegranate	3.62 ± 0.14
5	Treatment with metronidazole	3.54 ± 0.09
6	Treatment combination of pomegranate and metronidazole	1.7 ± 0.12

* zero count of *H. pylori.* Number of animals: *n* = 6 selected randomly from each group.

**Table 5:** Effect of *H. pylori* infection, PEAME, and metronidazole on the score of inflammation, metaplasia, and COX2 in the stomach mucosa of rats.

Scores 0–3	NC	PC	PEAME Proph.	PEAME Treat.	Met Treat.	PEAME + Met Treat
Inflammation	0	3	1	1	2	0
	0	3	2	0	1	1
	0	2	1	1	2	1
	0	2	0	2	1	0
	0	2	1	2	1	1
	0	3	2	2	1	0
Metaplasia	0	1	1	1	3	0
	0	2	2	2	1	0
	0	2	2	1	2	0
	0	2	0	1	2	0
	0	2	1	0	0	0
	0	1	0	0	0	0
COX2	0	3	2	2	2	1
	0	3	2	1	1	1
	0	3	2	2	3	1
	0	3	3	1	2	2
	0	3	2	2	2	2
	0	3	2	3	3	0

NC: negative control (not infected), PC: positive control (infected with *H. pylori*), PEAME proph.: infected rats taking PEAME as prophylactic, PEAME Treat.: infected rats treated with PEAME, Met Treat.: infected rat treated with metronidazole, PEAME + Met Treat: infected rats co-treated with PEAME and metronidazole.

## Data Availability

Not applicable.
